# The DynaSig-ML Python package: automated learning of biomolecular dynamics–function relationships

**DOI:** 10.1093/bioinformatics/btad180

**Published:** 2023-04-20

**Authors:** Olivier Mailhot, François Major, Rafael Najmanovich

**Affiliations:** Department of Biochemistry and Molecular Medicine, Université de Montréal, Montreal H3T 1J4, Canada; Department of Computer Science and Operations Research, Université de Montréal, Montreal H3T 1J4, Canada; Institute for Research in Immunology and Cancer, Université de Montréal, Montreal H3T 1J4, Canada; Department of Pharmacology and Physiology, Université de Montréal, Montreal H3T 1J4, Canada; Department of Computer Science and Operations Research, Université de Montréal, Montreal H3T 1J4, Canada; Institute for Research in Immunology and Cancer, Université de Montréal, Montreal H3T 1J4, Canada; Department of Pharmacology and Physiology, Université de Montréal, Montreal H3T 1J4, Canada

## Abstract

The DynaSig-ML (‘Dynamical Signatures–Machine Learning’) Python package allows the efficient, user-friendly exploration of 3D dynamics–function relationships in biomolecules, using datasets of experimental measures from large numbers of sequence variants. It does so by predicting 3D structural dynamics for every variant using the Elastic Network Contact Model (ENCoM), a sequence-sensitive coarse-grained normal mode analysis model. Dynamical Signatures represent the fluctuation at every position in the biomolecule and are used as features fed into machine learning models of the user’s choice. Once trained, these models can be used to predict experimental outcomes for theoretical variants. The whole pipeline can be run with just a few lines of Python and modest computational resources. The compute-intensive steps are easily parallelized in the case of either large biomolecules or vast amounts of sequence variants. As an example application, we use the DynaSig-ML package to predict the maturation efficiency of human microRNA miR-125a variants from high-throughput enzymatic assays.

**Availability and implementation:**

DynaSig-ML is open-source software available at https://github.com/gregorpatof/dynasigml_package.

## 1 Introduction

The Elastic Network Contact Model (ENCoM) is the only explicitly sequence-sensitive coarse-grained normal mode analysis model (Frappier and Najmanovich 2014). Its sequence sensitivity enables its use to predict the impact of sequence variants on biomolecular function through changes in predicted stability (Frappier and Najmanovich 2015) and dynamics (Teruel et al. 2021). We recently extended ENCoM to work on RNA molecules and predicted microRNA maturation efficiency from a dataset of experimentally measured maturation efficiencies of over 26 000 sequence variants using LASSO regression (Mailhot et al. 2022). To do so, the ENCoM Dynamical Signatures, which are vectors of predicted structural fluctuations at every position in the system, were used as input variables in a LASSO multiple linear regression model (Tibshirani 1996) to predict maturation efficiency. To our knowledge, this coupling of coarse-grained normal mode analysis to machine learning in order to predict biomolecular function as a result of the dynamical impact of mutations is the first of its kind. Here, we present the DynaSig-ML (‘Dynamical Signatures–Machine Learning’) Python package, which implements, automates, and extends that novel protocol. Considering that ENCoM can be used to study proteins, nucleic acids, small molecules, and their complexes (Mailhot and Najmanovich 2021), DynaSig-ML can be applied to any biomolecule for which there exist experimental data linking perturbations (such as mutations or ligand binding) to function. To demonstrate the use of DynaSig-ML, we predict maturation efficiencies of miR-125a sequence variants (Mailhot et al. 2022), exploring gradient boosting, and random forest (RF) regressors in addition to LASSO regression. An accompanying online step-by-step tutorial takes users through the necessary steps to generate all results shown in the present work. DynaSig-ML automatically computes the ENCoM Dynamical Signatures from a list of perturbed structures (mutations or ligand binding), stores them as lightweight serialized files, and can then be used to train machine learning algorithms using the Dynamical Signatures as input features. Any machine learning algorithm implemented by the popular scikit-learn Python package (Pedregosa et al. 2011) is supported as a backend for DynaSig-ML. In the case of LASSO regression or other forms of regression, the learned coefficients can be automatically mapped back on the studied structure by DynaSig-ML and visualized in 3D with two simple PyMOL (Delano 2002) commands. These coefficients represent the relationship between flexibility changes at specific positions and the predicted experimental property so the mapping can be used to drive new biological hypotheses (Mailhot et al. 2022). DynaSig-ML also automatically generates graphs showing the performance of each machine learning algorithm test. As mentioned, the necessary steps to apply DynaSig-ML are documented online as part of a step-by-step tutorial (https://dynasigml.readthedocs.io).

## 2 Implementation

DynaSig-ML runs the ENCoM model within NRGTEN, another user-friendly, extensively documented Python package (Mailhot and Najmanovich 2021). The machine learning models are implemented using the scikit-learn Python package (Pedregosa et al. 2011). The numerical computing is accomplished by NumPy (Oliphant 2006) and the performance graphs are generated with matplotlib (Hunter 2007), making these four packages the only dependencies of DynaSig-ML.

## 3 microRNA-125a maturation efficiencies

**Figure 1:**
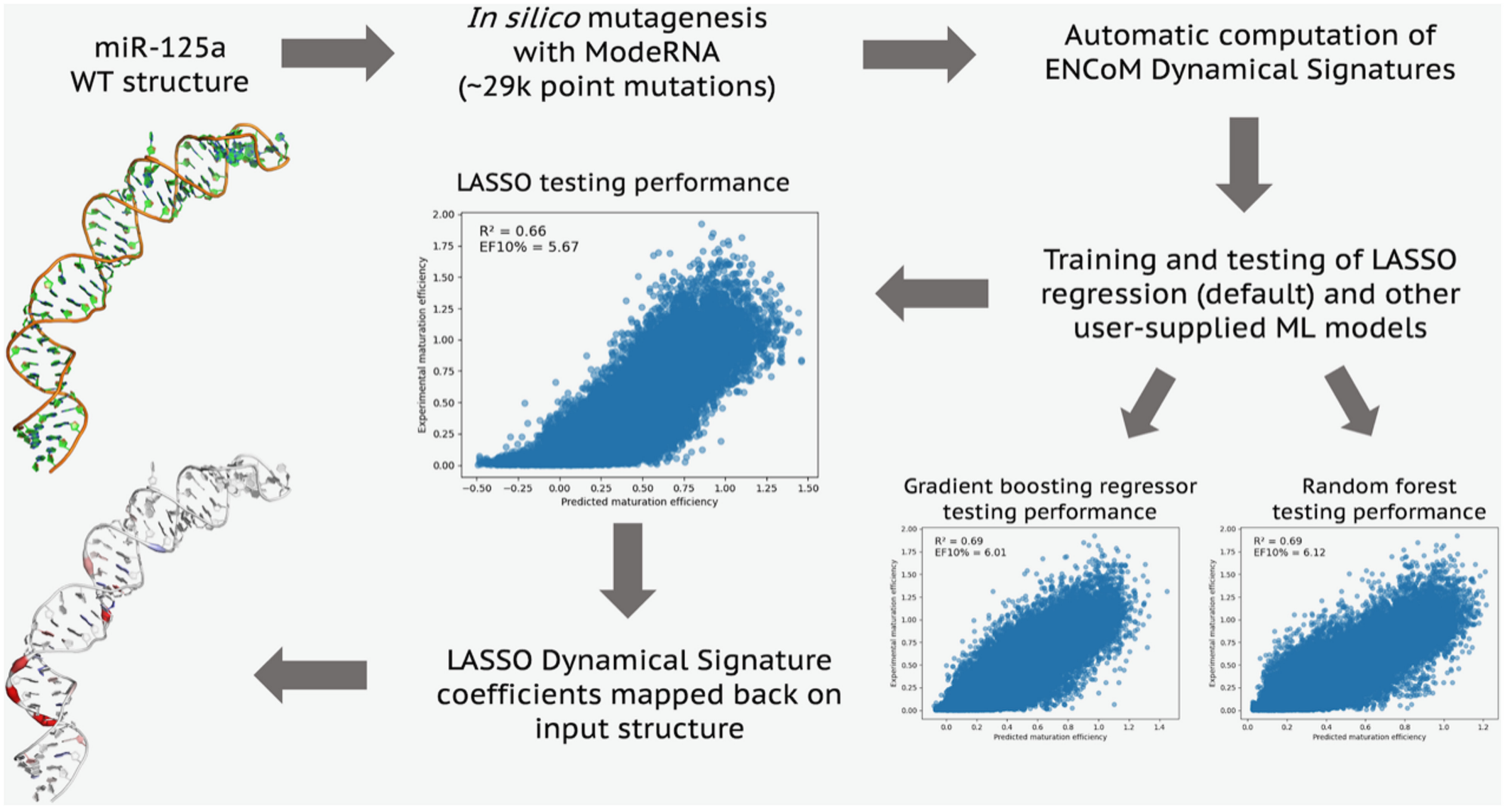
ENCoM-DynaSig-ML pipeline applied to miR-125a maturation efficiency data. The MC-Fold | MC-Sym (Parisien and Major 2008) predicted 3D structure of WT miR-125a is used as a template to perform the 29 477 point mutations with experimental maturation efficiency data using the ModeRNA software (Rother et al. 2011), all subsequent steps are performed using DynaSig-ML. For each of the *in silico* variants, a Dynamical Signature is computed with ENCoM. LASSO regression models with varying regularization strengths are trained by default, using as input variables the Dynamical Signatures and other user-supplied data (here, MC-Fold enthalpy of folding for each variant). Other ML models can be user-specified (here, gradient boosting regressor and random forest regressors). In the case of the LASSO regression model, the independence of the input variables allows the mapping of the learned coefficients back on the miR-125a structure. The color gradient represents each coefficient, from blue for negative coefficients, to white for null coefficients and red for positive coefficients. The largest absolute value coefficient will have the brightest color. The sign of a coefficient captures the nature of the relationship between flexibility changes at that position and the experimental property of interest (in this case, maturation efficiency). Negative coefficients mean that rigidification of the position leads to higher efficiency, while positive coefficients mean that softening of that position leads to higher efficiency. The thickness of the cartoon represents the absolute value of the coefficients, i.e. their relative importance in the model. In the present example, the positive coefficients on the backbone of base pairs 7, 9, and 11 identify the well-known mismatched GHG motif (Fang and Bartel 2015)

microRNAs are short single-stranded RNAs of ∼22 nucleotides which regulated gene expression by guiding the RNA-induced silencing complex to complementary regions within messenger RNAs. In our recent work, we adapted ENCoM to work on RNA molecules and used it to study dynamics–function relationships apparent from an experimental mutagenesis dataset (Fang and Bartel 2015) of over 29 000 sequence variants of miR-125a, a human microRNA (Mailhot et al. 2022). In order to illustrate a typical use case of DynaSig-ML, we applied it to study dynamics–function relationships in miR-125a sequence variants, replicating the results from our work in an automated way. Furthermore, we tested an RF model and a gradient boosting regressor as the machine learning backend of DynaSig-ML in addition to the default LASSO regression. Figure 1 illustrates the whole protocol used to start from the structure of WT miR-125a predicted with the MC-Fold | MC-Sym pipeline (Parisien and Major 2008), train the machine learning models, test their performance, and map the LASSO coefficients back on the miR-125a structure.

The results reported in Fig. 1 use our inverted dataset previously describes (Mailhot et al. 2022), in which the training set contains variants with only one or two mutations and the testing set contains variants with three to six mutations. It tests the models’ ability to generalize to variants containing more mutations than what was seen in training, which is very relevant in the context of using DynaSig-ML for high-throughput *in silico* predictions. However, this dataset does not exclude the possibility that no true dynamical signal is captured, and the models simply learn sequence patterns from their impact on the Dynamical Signatures. We developed a so-called hard dataset to answer this question and confirmed that a true dynamical signal is captured (Mailhot et al. 2022). A more in-depth analysis of the results for the three tested ML models, applied to both inverted and hard dataset and using all combinations of input variables (Dynamical Signatures and/or enthalpy of folding) can be found in the Supplementary Information. All results presented can be replicated by following the online DynaSig-ML tutorial and cloning the accompanying GitHub repository (https://github.com/gregorpatof/dynasigml_mir125a_example). When combining the enthalpy of folding and Dynamical Signatures, we obtain LASSO, gradient boosting (GBR), and RF models reaching respective testing performances of *R*^2^ = 0.66, *R*^2^ = 0.69, and *R*^2^ = 0.69. The enrichment factors at 10%, which are values ranging from 0 to 10 characterizing the relative proportion of the top 10% measured values in the top 10% predicted values, are 5.67, 6.01, and 6.12 for the LASSO, GBR, and RF models, respectively.

## 4 Conclusions

In conclusion, the DynaSig-ML Python package allows the fast and user-friendly exploration of dynamics–function relationships in biomolecules. It uses the ENCoM model, the first and only sequence-sensitive coarse-grained normal mode analysis model, to automatically compute Dynamical Signatures from structures in PDB format, stores them as lightweight serialized Python objects, and automatically trains and tests LASSO regression models to predict experimental measures, in addition to any user-specified machine learning model supported by scikit-learn. Moreover, DynaSig-ML automatically generates performance graphs and maps the LASSO coefficients back on the input PDB structure. A detailed online tutorial is available to replicate the miR-125a maturation efficiency application presented here (https://dynasigml.readthedocs.io).

## Supplementary Material

btad180_Supplementary_Data

## Data Availability

All data is in the supporting data file.
