# Supplementary material for: The DynaSig-ML Python package: automated learning of biomolecular dynamics–function relationships
Source: Bioinformatics. 2023 Apr 20;39(4):btad180. doi: 10.1093/bioinformatics/btad180 (PMC10130421; doi:10.1093/bioinformatics/btad180)
Supplement: btad180_Supplementary_Data [file btad180_supplementary_data.pdf]

Supplementary information for: The DynaSig-ML Python package: automated learning of biomolecular dynamics-function relationships

Olivier Mailhot[1−4] François Major[2,3] Rafael Najmanovich[4,*]

[1]Department of Biochemistry and Molecular Medicine, Université de Montréal, Montreal, Canada
[2]Department of Computer Science and Operations Research, Université de Montréal, Montreal, Canada
[3]Institute for Research in Immunology and Cancer, Université de Montréal, Montreal, Canada
[4]Department of Pharmacology and Physiology, Université de Montréal, Montreal, Canada
[*]To whom correspondence should be addressed.

February 24, 2023

Contact: rafael.najmanovich@umontreal.ca

1 SUPPLEMENTARY INFORMATION

1.1 *Training and testing datasets*

The results presented in Figure 1 (from the main text) used our previously published inverted dataset of miR-125a maturation efficiency [1]. The training set contains all miR-125a variants with at most 2 mutations, while the testing set contains all variants containing 3 to 6 mutations. This dataset represents a typical usecase of DynaSig-ML, where some experimental data (for instance, DMS data on a protein of interest) is known but the user is interested in predicting the effect of theoretical double or triple mutants.

However, while the performance of DynaSig-ML is high on the inverted dataset, it does not prove that a true dynamical signal is captured. Indeed, the models could be learning sequence patterns since all positions are mutated in the training set, and changes in sequence lead to changes in Dynamical Signatures through ENCoM's sensitivity to the all-atom context of the input structure. Investigating whether a true dynamical signal is captured was the motivation behind the development of our hard dataset. In that dataset, the middle base pair from each of the 8 mutated boxes (3 base pairs per box) is never mutated in the training set, and the testing set contains variants which only affect these specific base pairs. Thus, a model based on sequence alone captures exactly zero signal from the hard dataset, as we have previously shown [1].

1.2 *Results*

We report the testing performance of LASSO, gradient boosting (GBR) and random forest models when trained using the ENCoM Dynamical Signatures combined to the MC-Fold enthalpy of folding, the Dynamical Signatures alone, and the enthalpy of folding alone. These are reported both for the inverted dataset and the hard dataset. The testing performances on the hard dataset are shown in Supplementary Figure 1, Supplementary Figure 2 and Supplementary Figure 3 for the combined Dynamical Signatures and enthalpy of folding, the Dynamical Signatures alone and the enthalpy of folding alone, respectively. On the hard

dataset, GBR performs the best when combining Dynamical Signatures and enthalpy of folding, while it does not perform well when using these predictors on their own. This behavior could suggest a complementarity of these respectively entropy- and enthalpy-driven variables. When looking at LASSO regression, which is the easiest model to interpret, a smilar conclusion can be reached. Indeed, the LASSO model trained on Dynamical Signatures captures 9% of the variance, while the one trained on the enthalpy of folding alone captures 7% of the variance. When using the combination of both, 21% of the variance is captured, which is a sizeable increase over the 16% one would expect if the two types of predictor variables were simply additive.

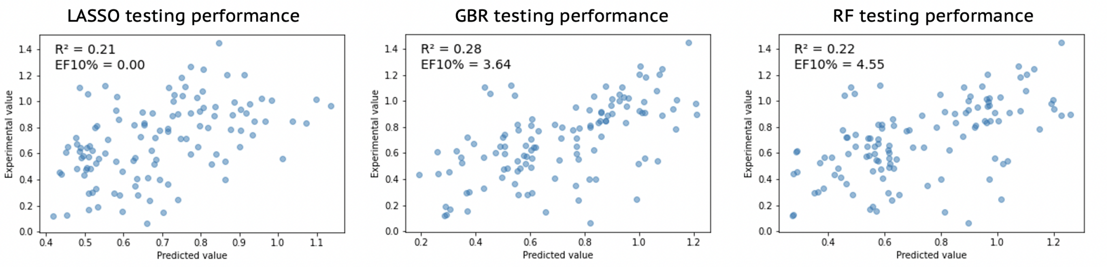

Supplementary Figure 1: Testing performance on the hard dataset using combined Dynamical Signatures and enthalpy of folding.

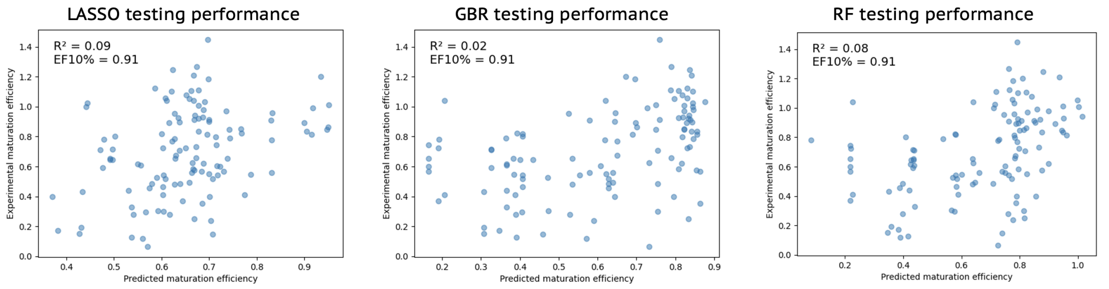

Supplementary Figure 2: Testing performance on the hard dataset using only Dynamical Signatures.

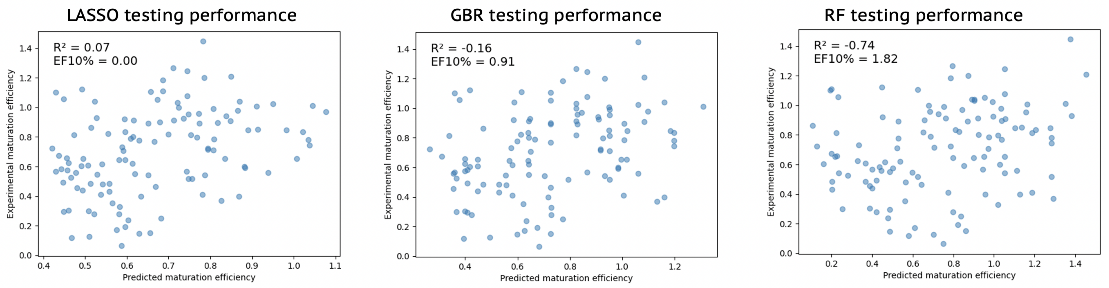

Supplementary Figure 3: Testing performance on the hard dataset using only enthalpy of folding.

The detailed results for the inverted dataset are presented in Supplementary Figure 4, Supplementary Figure 5 and Supplementary Figure 6, similarly as for the hard dataset. Here again, some complementarity is observed between the enthalpy of folding and the Dynamical Signatures. However in this case, it is less than additive, suggesting that when information about all positions is available there is redundancy in the signals captured.

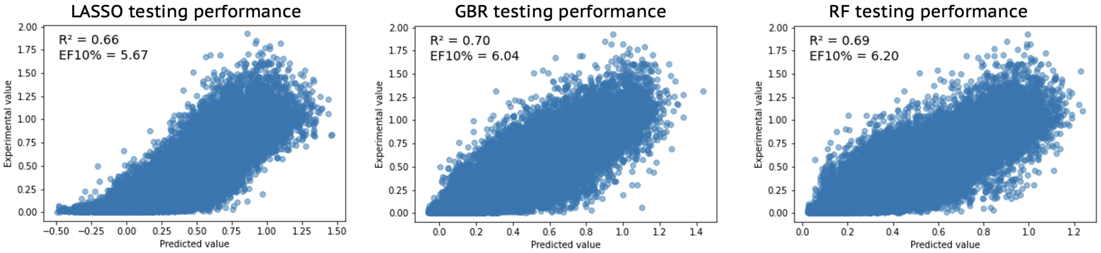

Supplementary Figure 4: Testing performance on the inverted dataset using combined Dynamical Signatures and enthalpy of folding.

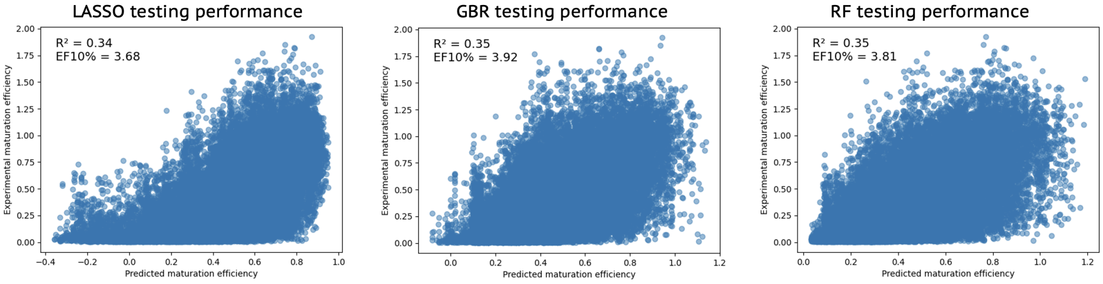

Supplementary Figure 5: Testing performance on the inverted dataset using only Dynamical Signatures.

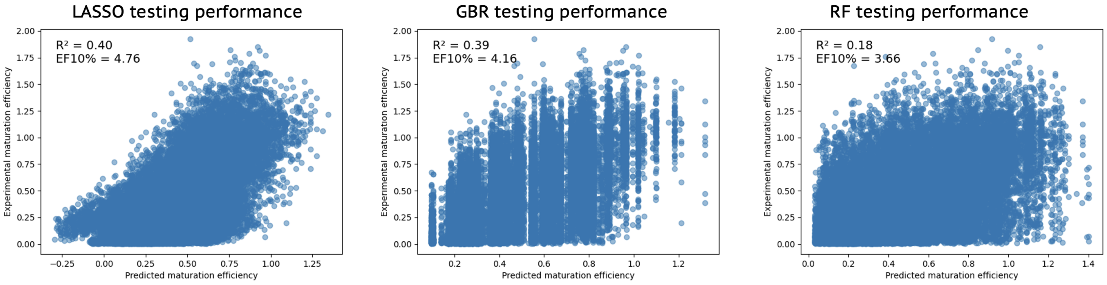

Supplementary Figure 6: Testing performance on the inverted dataset using only enthalpy of folding.

REFERENCES

[1] Olivier Mailhot, Vincent Frappier, François Major, and Rafael J Najmanovich. Sequence-sensitive elastic network captures dynamical features necessary for mir-125a maturation. *PLOS Computational Biology*, 18(12):e1010777, 2022.
